# Advances in Vaccine Adjuvants for Teleost Fish: Implications for Aquatic Welfare and the Potential of Nanoparticle-Based Formulations

**DOI:** 10.3390/vaccines12121347

**Published:** 2024-11-28

**Authors:** Iosif Tammas, Konstantina Bitchava, Athanasios I. Gelasakis

**Affiliations:** 1 Laboratory of Applied Hydrobiology, Department of Animal Science, Agricultural University of Athens, 11855 Athens, Greece; stud217095@aua.gr; 2 Laboratory of Anatomy & Physiology of Farm Animals, Agricultural University of Athens, 11855 Athens, Greece

**Keywords:** aquaculture, vaccines, adjuvants, nanoparticles, immunization, inflammation, granulomas, lesions, sustainability, welfare, health, side effects, teleost fish

## Abstract

Vaccine adjuvants are crucial for reinforcing the immunogenicity of vaccines. Therefore, they are widely used in the aquaculture sector as vaccine components, facilitating the efficient prevention of infectious diseases and promoting sustainable teleost fish growth. Despite their benefits, there has been a growing concern about the potential adverse effects of vaccine adjuvants in teleost fish, connoting a valid impact on their overall health and welfare. Among the adjuvants used in aquaculture vaccinology, nanoparticle-based formulations have given rise to a promising new alternative to traditional options, such as oil-based emulsions and aluminum compounds, offering the benefit of minimizing relevant side effects. The aim of this paper was to review the current status of the adjuvants used in aquaculture, provide a description and an evaluation of their mode of action and side effects, and explore the potential use of nanoparticle formulations as adjuvants to improve the efficacy of aquaculture vaccines. By demonstrating and assessing the equilibrium between teleost fish welfare and immunological efficacy, this review presents a collective perspective that will assist in establishing a framework for the utilization of effective species-specific practices around adjuvant use in aquaculture, while also addressing the challenges of welfare-friendly immunization.

## 1. Introduction

In the dynamic landscape of aquaculture, the use of vaccines has emerged as a cornerstone for disease prevention, marking an essential checkpoint in sustainable industry growth [1]. Vaccines constitute the most efficient solution to combat impeding pathogen threats, especially in the context of modern aquaculture, where increased rearing densities have caused a spike in microbial agent transmission [2,3]. The ecological pressure exerted by aquaculture practices in the global transmission of pathogens in wild fish populations has added an additional layer to the issue, proving that the efficient and timely development of vaccines is paramount in safeguarding the health of teleost fish species [4,5,6].

To further boost the immunogenicity and the elicitation of robust immune responses, auxiliary substances called adjuvants have long been used in aquaculture vaccine formulations to maximize the effectiveness of the administered vaccine preparations. Traditionally, these substances were abstractly described as agents that either improved vaccine potency or efficiency, two terms that were used to signify boosts to adaptive immune responses and the prevention of infection, respectively [7]. Considerable advancements in the field of immunology, however, led to a novel, clearer, and more refined term for adjuvants, defining them as structurally heterogenous compounds that regulate the inherent immunogenicity of vaccine antigens [7]. This new definition acknowledges the role of adjuvants in boosting immunizations through specific and intricate mechanisms of action, thereby consolidating a promising field for scientific exploration in the field of immunology.

The use of adjuvants in aquaculture is compatible with every method of vaccine administration, including injection, immersion, and oral vaccination. The vast majority of vaccines utilized in aquaculture, however, are administered via intraperitoneal (IP) injection, since this method of vaccine delivery provides the most adequate and long-lasting immunization in teleost fish [7,8,9]. Nevertheless, the use of specific adjuvants has enabled other vaccine delivery methods, such as immersion vaccination, to be efficiently utilized in aquaculture, thus opening the gateway for alternative methods of vaccine administration. By enhancing the effectiveness of alternative vaccination methods, the industry can potentially shift from the exclusive use of injection vaccination to other forms of vaccine delivery that will offer benefits not only in logistical and cost-related aspects, but also in animal welfare, by reducing the stress induced during the immunization process.

In this quest for innovative and sustainable immunization strategies, nanoparticles have paved the way for a promising alternative to traditional adjuvant formulations in the aquaculture sector. Thanks to their small size and their ability to provide tailored platforms for controlled release, these formulations offer enhanced antigen delivery and improved immune response, while minimizing the required dosages and mitigating any relevant side effects associated with traditional adjuvants [10,11,12]. Recent data suggest that nanoparticles not only boost the efficacy of conventional injectable vaccines, but also improve immunization methods that involve mucosal delivery, such as immersion and oral vaccination [13]. By promoting enhanced antigen uptake and by stimulating both innate and adaptive immunity, nanoparticle-based adjuvants can revolutionize vaccine protocols in aquaculture, potentially reducing the reliance on invasive methods in favor of more cost-efficient and welfare-friendly routes. Furthermore, their capacity to encapsulate and protect antigens improves vaccine stability, prospectively broadening their use across various aquatic species and environmental conditions [14].

This review article aims to synthesize a comprehensive perspective on the most popular adjuvants currently used in aquaculture vaccinology, drawing information from literature not only about their exact modes of action, but also about advances made in understanding and mitigating their side effects. Additionally, the potential of utilizing nanoparticle formulations as an alternative to traditional vaccine adjuvants in the industry of aquaculture is discussed, offering a complete overview of the future directions for optimizing vaccine safety and enhancing immune responses. Spreading research findings regarding adjuvant side effects across different and diverse combinations of adjuvants, vaccines, fish species, and rearing conditions is paramount in broadening the knowledge on the assessment of these adverse effects, thereby safeguarding aquatic organism health and sustainability in practice. Delving deeper into the matter, understanding exactly how adjuvants work is essential for unraveling their importance and integration in vaccination strategies and their implications for teleost fish welfare.

## 2. Mode of Action: The Two-Signal Model

The precise mechanisms by which adjuvants enhance immune responses have long been, and continue to be, a subject of significant debate and investigation. While adjuvants are known to enhance the body’s immune response to antigens, the detailed processes by which they achieve this remain elusive [15,16]. Researchers have proposed many assumptions, including the stimulation of innate immune receptors, the creation of a “depot effect” that prolongs antigen exposure, and the modulation of the immune environment; yet, a complete understanding is still lacking, especially in the context of aquatic organisms [7]. In this quest for understanding how adjuvants work, Schijns’ model, also known as the two-signal model [17], stands out as the most comprehensive model for explaining and categorizing their mechanisms of action, providing a structured approach to these complex interactions, while highlighting the need for continued research to fully unravel the intricacies of adjuvant function to improve vaccine development. According to this proposed model, vaccine adjuvants can be categorized based on their capacity to facilitate two types of signals: type 1 and type 2 signals.

### 2.1. Type 1 Signals

Type 1 signals typically refer to the process of antigen presentation by specific types of immune cells, called professional antigen-presenting cells (pAPCs) [7]. These cells can bind to antigens through highly polymorphic MHC class II proteins expressed on their surface and subsequently display them to T-cells to facilitate their differentiation into CD4+ T-cells, known as helper T-cells or T_h_ cells. This sequence of events is essential not only to the production of antibodies, but also to the induction of innate immune responses, since T_h_ cells can aid by providing necessary co-stimulatory signals that are unique to the type of antigen that is recognized [18,19,20,21]. Several immune cells are known to possess antigen-presenting functions, including B-cells, macrophages, and dendritic cells (DCs), and this is a pattern that is conserved in teleost fish, albeit with some differences [22].

Adjuvants that influence type 1 signals and antigen presentation thereof can be categorized in a distinct group that encompasses type 1 signal facilitators that can essentially catalyze the antigen presentation process. The exact way in which type 1 adjuvants influence can vary; however, it all boils down to improving the immunoavailability of the vaccine’s antigens to antigen-presenting cells [16]. Based on that, and by taking the geographical concept of immune reactivity into consideration [23], adjuvants that have a significant effect on an antigen’s presentability in the context of space, time, and availability can collectively be categorized as type 1 adjuvants according to the two-signal model.

Although type 1 adjuvants are widely implemented in vaccinology and are considered crucial for eliciting robust immune responses, the precise mechanisms behind their effects remain largely elusive at present [16]. Since type 1 adjuvants are used extensively in injectable vaccines, the hypothesis for the most important mechanism of action thus far proposes that these adjuvants facilitate a “depot effect”, in which a localized reservoir of the antigen is formed at the site of injection [7,17]. This allows for a controlled, gradual, and sustained release of the antigen over an extended period of time, therefore affecting its overall immunoavailability to the host’s immune system cells [16]. By maintaining the antigen’s presence in the body for a longer period, the depot effect maximizes the immune system’s exposure to the vaccine’s antigen, thereby promoting a more robust and prolonged immune response.

The evidence on whether the “depot effect” is solely responsible for the immunostimulating effects of type 1 adjuvants or whether it simply affects it remains debatable, keeping many researchers conflicted on the topic [24]. Many studies imply that the tissue microenvironment of the injection site plays an important role in immunostimulation, as many antigen-presenting cells are attracted to chemotactic factors produced locally at this site [16,24]. Many key players of the innate wing of the immune system enter the injured tissue and influence the function of APCs. Based on that, it appears that type 1 adjuvants can potentially promote inflammatory signals, adding an additional layer of complexity to the modes of action supporting their immune-enhancing effects.

### 2.2. Type 2 Signals

On the other hand, type 2 signals, according to the two-signal model, are regarded as secondary, ancillary signals that facilitate the activation of adaptive immune cells like B-cells and T-cells [7,16,17]. These signals provide necessary co-stimulation during the process of antigen presentation, which is fulfilled through the equilibrated and combinatory effects of co-stimulatory or co-inhibitory signals [16]. These signals can thus tailor the scale and the effect of adaptive immune responses, resulting in varying outcomes of immunostimulation that aid in enhancing the process of immunization. As such, adjuvants that facilitate type 2 signals can collectively be grouped into type 2 adjuvants.

The activity of adjuvants that facilitate type 2 signals has been well described, even down to the molecular level, as opposed to those that facilitate type 1 signals [16]. The cells of the innate immune system express pattern-recognition receptors (PRRs) that are used to recognize pathogens by binding to specific pathogen-associated molecular patterns (PAMPs) [16]. These molecular patterns are typically structural elements that are unique to different classes of pathogens and are recognized by different kinds of PRRs [25,26]. The recognition of pathogen-associated molecular patterns by the receptors of the innate immune cells orchestrates signaling cascades that facilitate the induction of both humoral and cellular immune responses. Some of the most important examples of pattern-recognition receptors’ subfamilies and their associated ligands identified so far in teleost fish are summarized in Table 1.

Type 2 adjuvants facilitate the stimulation of the immune system by simulating the recognition of PAMPs by specific pattern-recognition receptors. However, another way by which type 2 adjuvants can stimulate the innate immune system lies in the induction of the so-called danger signals that are associated with tissue injury and damage. According to the model proposed by Matzinger, the immune system does not necessarily focus on the recognition of microbial agents by themselves, but rather on the co-recognition of antigens associated with danger signals [42]. Upon entry and infection, microbial agents cause damage and stress to surrounding tissues, enabling the induction of signals that are materialized through the release of damage-associated molecular patterns, or DAMPs. These patterns can include both segments of dead cells and molecules like heat-shock proteins, uric acid, and cytokines that promote the activation of stimulatory signals for the pAPCs [16]. According to Ribeiro and Schijns, this can elucidate the mechanism of action behind several type 1 adjuvants as well, adding an interesting element of overlapping between the immunostimulating machineries involved in both type 1 and type 2 adjuvants.

Overall, it is accepted that the categorization of adjuvants to type 1 or type 2 facilitators is not necessarily mutually exclusive [16]. The intricate mechanisms behind the activation of signaling cascades that modulate immune responses can be affected in numerous ways, highlighting that the study of different adjuvants is important in the elucidation of the interplay between immune-enhancing compounds and the elements of the immune system. Adjuvants that facilitate type 2 signals can influence a plethora of immunostimulating pathways, and that is evidenced by the fact that one of the most well-studied PRR receptors, the toll-like receptors (TLRs), can aid in the activation of both innate and adaptive immunity [16], bridging the gap between the two immune subsystems. Upon binding to pattern-recognition receptors, different molecules can have different outcomes in terms of immune-enhancing capabilities, thus facilitating the induction of different stimulatory signals that are materialized through the secretion of cytokines [26,27,28,43].

### 2.3. Further Elaborations on the Two-Signal Model

Since the binding of PRR ligands precedes the secretion of cytokines and co-stimulatory molecules, it has been suggested that the binding of PAMPs to the PRRs constitutes a separate type of signal, often referred to as a type 0 signal [16]. According to this proposal, this type of signal facilitates the initiation of all down-stream pathways involved in the secretion of co-stimulatory signals that make up the type 2 signal category, ultimately making a distinction between the effects of PRR ligand binding and the stimulatory effects of type 2 signals on the activation of naive B-cells and T-cells. This distinction can potentially aid in advancing the field of adjuvant immunology by allowing research to focus on adjuvants that have profound stimulatory effects on each type of signal. This can include studies on PRR agonists co-delivered with antigens, inhibitory molecules that block immune-attenuating signals, and recombinant co-stimulatory molecules that effectively emulate the immune-amplifying capabilities of endogenous cytokines directly [16].

In a recent update of events, further elaborations on Schijns’ proposed model appear to have been made, making the case for a fourth type of signal (type 3 signal) that regulates the proliferation of T-cells into different antigen-specific subpopulations, as with regulatory T-cells (Tregs) and different subsets of T_h_ cells [44]. Additionally, a fifth type of signal called a type 4 signal can be used to describe adjuvants that affect the localization of vaccine antigens by directing them to specific anatomical regions within the host’s body [44]. This aspect adds another source of complexity to the activity of adjuvants, as vaccine formulations need to not only activate the immune system, but also ensure the correct direction of immune effector cells and the induced immune response. In aquaculture vaccinology, this can be especially important for vaccines targeting mucosal routes, like the skin, the gills, and the intestines.

**Figure 1:**
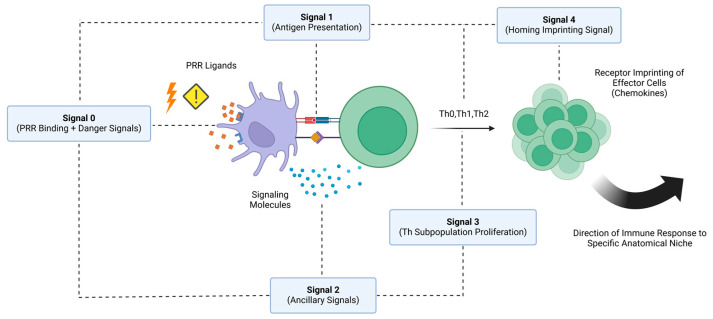
An overview of all elaborations made in the two-signal model to effectively explain the mode of action behind several vaccine adjuvants. An effective immunization is usually the result of a well-orchestrated immune response, and thus, the synergistic effect of antigens and vaccine adjuvants facilitating certain signals can aid in eliciting a robust and long-lasting protective effect.

The mucosal surfaces are primary entry points for many aquatic pathogens [45,46,47]. By potentially utilizing adjuvants that enhance the mucosal imprinting of type 4 homing signals, vaccines can be designed to induce robust and localized immune responses that will reduce disease incidence and improve overall teleost health and aquaculture sustainability. This lies in accordance with the current direction of aquaculture vaccinology that aims to develop vaccines that will not only be able to elicit mucosal immunity, but also be administered through routes that are more preferrable to the industry, such as immersion and oral vaccination [1]. Effectively, this can also reduce the stress induced on fish during the vaccination process, therefore promoting their overall welfare. A schematic overview synthesizing the most recent insights incorporated into the signal modeling of vaccine adjuvant activity can be seen in Figure 1.

## 3. Traditional Adjuvants Used in Aquaculture Vaccinology

**Figure 2:**
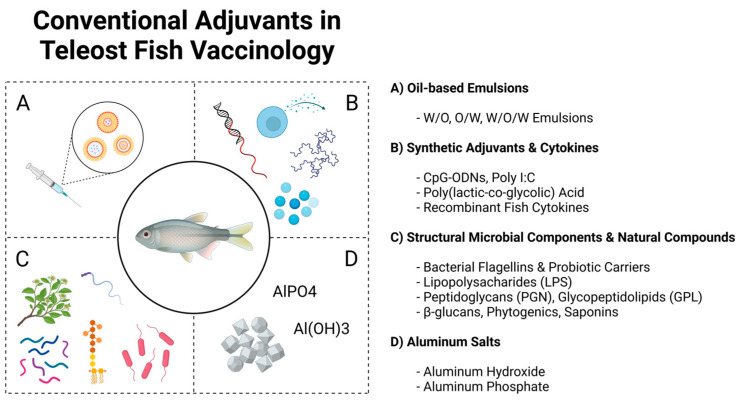
A schematic illustration showcasing the main types of vaccine adjuvants used conventionally so far in teleost fish vaccinology.

In the realm of aquaculture vaccinology, adjuvants are key components that enhance the immunogenicity of vaccines, which makes them indispensable in the development of efficient vaccines for aquatic species. This section will delve into the major adjuvants currently employed in aquaculture vaccines, exploring their modes of action, efficacy, and their influence on immune cell activation and homing. By gaining a deeper grasp on the unique properties and applications of these adjuvants, a better understanding of their potential exploitation can be established, leading to the improved assessment of their side effects and their mitigation thereof in different fish species. An illustration summarizing the main adjuvants discussed below can be seen in Figure 2.

### 3.1. Oil-Based Emulsions

Oil-based emulsions are widely used in aquaculture vaccine formulations, especially due to their proven efficacy in enhancing immunization [48]. Emulsions consist of a dispersed liquid distributed inside a second liquid, forming two phases, the continuous and the dispersed, where typically the two liquids would not be able to mix naturally. In vaccine formulations, these phases typically consist of an aqueous continuous phase, loaded with the vaccine antigen, and an oily phase that is dispersed [7]. The formation of the emulsion is facilitated by the use of emulsifiers that act as surfactants, stabilizing the emulsion by reducing the surface tension between the two liquids thanks to their polar groups, which are hydrophilic, and their non-polar groups, which are hydrophobic [7,48]. Surfactants can be categorized by taking into consideration the value of the analogy of the hydrophilic to the hydrophobic groups, known as hydrophilic–lipophilic balance (HLB), a term first proposed by Griffin in 1949 [49].

The HLB value of surfactants is a determining factor for the production of different types of emulsions [49,50,51] that have different efficiencies as vaccine adjuvants [7]. Emulsifiers with low HLB values lead to the creation of water-in-oil emulsions (W/O), whereas those with high HLB values tend to form oil-in-water emulsions (O/W) that are typically better tolerated but exhibit weaker immune responses [7]. Certain surfactant systems with an intermediate HLB value allow for the creation of W/O/W emulsions, where the continuous phase is aqueous and the dispersed phase is oil, within which an entrapped aqueous phase containing water-soluble or suspended antigens is found. This third type of emulsion has been shown to elicit robust immune responses with a variety of antigens, as summarized by Dalmo et al. [7].

The majority of aquaculture vaccines rely on adjuvants that are based on mineral oil emulsions [7,52]. The most common example of such adjuvants consists of Freund’s Complete Adjuvant (FCA), which is a mineral oil-based formulation used to create a W/O emulsion with the addition of a surfactant and heat-killed *Mycobacteria* [7,52]. Despite being effective, however, this adjuvant has raised concerns due to its association with severe side effects during administration, making its implementation restricted to mainly experimental settings, while gradually being replaced by Freund’s Incomplete Adjuvant (FIA), which lacks the mycobacterial elements [7]. Montanide adjuvants are a series of commercially available adjuvants based on various oil emulsions formulated by the company SEPPIC and licensed under the common trade name of Montanide^TM^. These adjuvants are not exclusively mineral oil-based, but their use has been well-established in the realm of veterinary vaccines, constituting the adjuvant of choice for many commercially available vaccines alongside other mineral oil-based adjuvant brand series in the market. A list of the currently commercially available Montanide^TM^ series formulations intended for use in teleost fish can be read below in Table 2.

Some of the most commonly used Montanide^TM^ adjuvants in aquaculture vaccines include the Montanide™ ISA 763A and 763B VG series, whose efficiency has been highlighted in numerous publications dealing with different species of fish and pathogens [53,54,55,56]. However, in accordance with evidence provided by the recent elaborations on the two-signal model, recent studies appear to suggest that the use of different adjuvants, including even commercialized ones, with different vaccine antigens, can result in a totally different type and scale of immunization [57,58,59,60]. This further strengthens the notion that the appropriate selection of the adjuvant–antigen combination is paramount in ensuring a robust and long-lasting immune response upon vaccination in the context of aquatic organisms, alluding to the fact that more research should be focused on such combinations to shed light into the optimization of vaccine formulations.

### 3.2. Aluminum-Based Compounds

Another category of popular vaccine adjuvants for aquaculture vaccines are aluminum compounds that contain aluminum salts, such as aluminum hydroxide or aluminum phosphate, collectively referred to as alum [7]. The mechanism of action of these adjuvants remains elusive, though quite a number of mechanisms have been proposed to explain their immune-enhancing capabilities [15,61]. Similarly to oil-based emulsions, it has been suggested that alum provides a “depot” effect by adsorbing the vaccine antigen onto its particles and creating an antigen reservoir at the site of administration [61,62,63,64]. Studies, however, have shown that an antigen depot is not necessary for the adjuvanticity of alum [63], alluding to the fact that there might be other mechanisms at play.

Today, it is strongly suggested that alum has a profound effect on phagocytosis, inflammation, dendritic cell maturation, and the secretion of cytokines [62,63]. Several studies have highlighted its role in the activation of the NLRP3 inflammasome, yet the definite mode of action is still under debate, as there is no universally established standard as far as immunization protocol, type of aluminum adjuvant, and animal model are concerned [62]. Alum does not appear to have the ability to influence dendritic cells directly through TLR binding pathways, though some level of interaction has been reported via TLR-independent binding to lipid cues in the dendritic cell membrane [63]. Additionally, alum has been shown to promote the induction of endogenous danger signals and facilitate the activation of the complement system, with the primary characteristic of its adjuvant activity lying in the generation of Th2-mediated humoral immune responses, as summarized by He et al.’s review [63].

The validation of these hypotheses and the elucidation of the intrinsic machinery involved in alum’s adjuvant action remains an interesting and elusive topic for researchers so far. In the context of aquatic organism immunology, the scarcity of information makes understanding the action of aluminum-based adjuvants additionally challenging; nevertheless, recent studies have shown that alum is a potent adjuvant in teleost fish, able to enhance immune responses upon vaccination. In Angosto et al.’s recent study, alum was shown to trigger the expression of the gene encoding IL-1β in both the European seabass (*Dicentrarchus labrax*) and gilthead seabream (*Sparus aurata*), matching the hypothesis that aluminum-based adjuvants can promote the secretion of inflammatory cytokines by activating the NLRP3 inflammasome [65]. Surprisingly though, no IL-1β secretion was observed in seabream leukocytes and macrophages, alluding to the fact that alum’s adjuvant action might be independent of the inflammasome in teleost fish, as further strengthened by the lack of conservation of caspase-1 processing sites in non-mammalian IL-1β, the inability of caspase-1 to modify IL-1β, and the failure of caspase-1 activation through NLRP3 inflammasome facilitators previously reported in gilthead seabream [66,67,68].

Despite the forementioned findings, alum remains a strong adjuvant in aquaculture vaccinology, able to facilitate the production of reactive oxygen species through a NADPH oxidase-mediated route [65]. Adding to the fact that alum crystals can potentially induce endogenous signals like uric acid and release DNA from dead cells [69,70,71], these results tilt towards the validation of the notion that alum’s adjuvant capacity is achieved through the induction of danger signals in the host’s body. However, the existence of side effects can also be attributed to alum’s immunostimulating capacity, as inflammatory reactions, granulomas, melanizations, and toxicity in the splenic melanomacrophage centers have been documented with alum use in teleost fish [72].

### 3.3. Synthetic Adjuvants and Cytokines

Synthetically derived adjuvants represent a unique category of adjuvants that have been facilitated by the relatively recent advent of scientific and technological innovation. In the realm of aquaculture, one of the most popular adjuvants of this type explored consists of synthetic oligodeoxynucleotides (ODNs) that express unmethylated motifs known as CpG. Such motifs are abundant in microbial DNA and can be recognized by immune cells expressing TLR9 [7], though recent reviews also portray TLR21 as a receptor [27,28]. Synthetic double-stranded polyribonucleotides called Poly I:C (Polyinosinic–polycytidylic acid) have also been used as adjuvants in aquaculture vaccinology, particularly to combat pathogens of viral origins. Their mode of action relies on simulating a viral infection, leading to the production of antiviral cytokines like type 1 interferons (IFNs), through binding to the TLR3 receptor [7]. However, as mentioned previously, recent data have shown that Poly I:C can also act as a ligand to the TLR22, TLR26, and TLR28 receptors in teleost fish, opening the possibilities for additional signaling pathways to be involved in the adjuvanticity of Poly I:C.

The exploration of fish cytokines as potential vaccine adjuvants in aquaculture was recently summarized in our previous paper [1], incorporating insights from Guo and Li’s review on the topic [73], along with findings from several recent studies highlighting their effects. Overall, recombinant cytokines and cytokine genes incorporated into vectors have shown promising results when incorporated as adjuvants into vaccine formulations. Although in its infancy, this approach has recently garnered attention, as cytokines can modulate immune responses and provide certain immunopotentiation effects during fish vaccination, without the danger of a strong generalized response and potential side effects [74]. Th0-derived cytokines like IL-1β, IL-8, IL-12, IL-15, IL-17, G-CSF, and TNF-α have been shown to have potential adjuvant activity in various teleost fish species and in combination with various pathogens and vaccine technologies, as is the case with Th1-derived IFN-α, IFN-γ, IFN-c, IL-2, and Th-2 derived IL-6 and β-chemokines [1,73,74].

Lastly, for oral vaccination, synthetic and biodegradable poly (lactic-co-glycolic) acid polymers, known simply as PLGA, have long been prevalent as vaccine carriers and potential adjuvants in teleost fish vaccination. Even though the vast majority of studies have explored PLGA microparticles as a vaccine delivery carrier through the method of antigen encapsulation, there have been a few reported publications that exhibit PLGA’s adjuvant activity as per Dalmo et al.’s review [7]. Since then, recent reports have successfully implemented PLGA-mediated delivery of DNA [75] and inactivated vaccines [76] in teleost fish immunization protocols, showcasing PLGA’s ability in conferring increased levels of protection against both viral and bacterial pathogens. In an exciting and promising new series of events, recent studies combining PLGA microparticles with cytokines [77] and CpG-ODNs [78] as adjuvants have also surfaced, demonstrating that synthetic adjuvants remain a fertile area of research in the future of aquaculture vaccinology, since these combinations show better immunization efficacy when compared to using each component individually.

### 3.4. Structural Microbial Components and Natural Compounds

Naturally occurring substances have similarly been explored for their adjuvant and immunostimulating capabilities in teleost fish immunization. Typically, this category encompasses structural components of microorganisms that exhibit promising adjuvant effects, since they constitute distinctive pathogen-associated molecular patterns and can act as PRR ligands for the host’s immune cells. Prime examples include lipopolysaccharides (LPSs), flagellins, β-glucans, and peptidoglycans (PGNs), which have been implemented as both feed immunostimulants and as adjuvants in aquaculture. These macromolecules induce the secretion of signaling cytokines and potentiate immune responses, opening the gateway for their application as both functional feed additives and vaccine adjuvants in a wide array of fish species, as evidenced by numerous publications on the topic [7,79,80,81,82,83,84]. Similarly, lipopeptides have been successfully used as vaccine adjuvants in fish, since they are abundant in many microbial species, including *Mycobacteria* and *Mycoplasma*. Polar glycopeptidolipids (pGPLs) of mycobacterial origin have shown promising immune-enhancing capabilities in fish, without the presence of any adverse side effects [85].

Besides PAMPs, however, other naturally occurring substances have exhibited promising results when used as potential aquaculture vaccine adjuvants. Saponins are a naturally occurring group of steroid or terpenoid glycosides that are typically extracted from plants. They have been shown to enhance innate immune responses in many animal species, including teleost fish and aquatic invertebrates [86]. In aquaculture vaccinology, saponins extracted from the soapbark tree (*Quillaja Saponaria*) have given rise to a promising vaccine adjuvant, known as Quil-A. The implementation of saponins as aquaculture vaccine adjuvants, however, has been met with adversity, as they can be toxic in high doses, and their stability is compromised in aqueous solutions [7,87]. Nevertheless, recent studies show that saponins can effectively augment immune responses, even when administered together with other adjuvants or through mucosal routes [88,89,90]. Additionally, several other plant-derived saponins, like ginseng stem leaf saponins, have successfully been reported to enhance immune responses in fish upon administration, as seen in recent studies [91,92].

The adjuvant capacity of several phytogenic substances for aquaculture vaccinology has been well described in a review recently published by Soltani et al. [93]. Furthermore, probiotics appear to gain traction as adjuvants in aquaculture vaccines, especially in oral vaccination applications dealing with encapsulation platforms. Coupled with modern genetic bioengineering techniques, probiotic bacteria like Bacilli, Lactobacilli, and Lactococci can have profound effects on fish immunization, opening the gateway for interesting vaccine delivery applications by combining the immune-stimulating effects with improved antigen delivery through the oral route [94,95,96,97,98,99,100,101,102,103]. Finally, the use of chitosan, the deacetylation product of the naturally occurring polysaccharide chitin derived from insects, fungi, and aquatic invertebrates, has been met with promise in the realm of aquaculture vaccinology, as it can be similarly used in oral routes as an immune-enhancing vehicle for vaccine delivery [104,105,106]. The adjuvant capacity of chitosan microparticles can be traced in a plethora of recent publications, both by itself, and in combination with other vaccine adjuvants or polymers [107,108,109,110,111,112,113,114,115].

## 4. Assessing the Side Effects of Traditional Adjuvants

**Figure 3:**
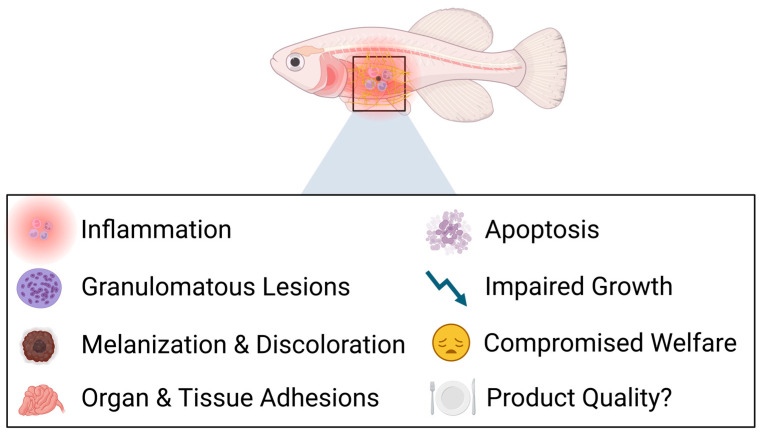
Potential side effects of the current adjuvanted injectable vaccines in teleost fish. In severe cases, the physiological functioning of the digestive system and thus the growth rate can be impaired, potentially undermining animal welfare and lowering the quality of aquatic food products.

Despite vaccine adjuvants being crucial components in enhancing the efficacy of aquaculture vaccines, it is well recognized that their use can be accompanied by several side effects and drawbacks. Oil-based adjuvants are currently dominating formulations used in aquaculture, as they are predominantly implemented in vaccines administered by way of intraperitoneal injection [8,116,117,118]. However, these vaccines are often associated with adverse side effects, stemming primarily from inflammatory reactions that are triggered during immunization. These reactions can exhibit varying degrees of intensity and severity, and the most typical symptoms include adhesions of tissues and organs to each other or to the peritoneal cavity, granuloma formation, and discoloration at the site of injection [117,118,119,120,121,122,123,124,125]. A schematic illustration summarizing the adverse effects of currently employed vaccine formulations in teleost fish can be seen below in Figure 3.

The Spielberg scale, developed in 1996 by Midtlyng et al., was designed to assess the severity of intraperitoneal symptoms in Atlantic salmon (*Salmo salar*) following immunization against furunculosis [126]. Remarkably, this scale and its underlying methodology remain valid and therefore prevalent in the international literature, and are particularly referenced in studies examining the side effects of vaccines in teleost fish of similar species. In this framework, individual fish are evaluated based on the severity of their symptoms and the associated pathological changes post-vaccination, by implementing a scale that typically ranges from 0 to 6; a higher score indicates more pronounced post-vaccination pathological findings, offering a tool for establishing a standardized approach in evaluating the impact of adjuvanted vaccines in teleost fish health. Recently, in 2021, Tziouvas and Varvarigos adapted a Spielberg-type scale to the European sea bass (*Dicentrarchus labrax*) [125]; a side-by-side adaptation comparison of the two scales can be seen in Table 3.

This study involved the collection of data from fish growing in Greek aquaculture sites that were vaccinated through intraperitoneal injection with licensed oil-adjuvanted vaccines over a three-year period (2016–2018) and the meticulous evaluation of the pathological findings found within the peritoneal cavity. As expected, many of the sampled fish exhibited the side effects described in the literature, such as chronic peritonitis, granulomatous lesions, discolorations, and tissue and organ adhesions. Notably, smaller-sized fish appeared to experience more severe side effects following vaccination, potentially due to the challenges associated with the administration method of IP injection, which requires careful handling and execution. Furthermore, vaccines formulated with mineral oil adjuvants that are not easily metabolized by fish, such as paraffin oils, resulted in more pronounced symptoms associated with intense inflammatory responses. The feed conversion rate (FCR) remained largely unaffected in the aquaculture sites, except in cases where there were severe granulomatous lesions and strong tissue adhesions hindering the physiological functioning of the digestive system.

The findings from the previous study, combined with simple macroscopic and histological analyses conducted during the autopsies, led to the development of a scale similar to that of Midtlyng et al. to estimate the severity of symptoms post vaccination. The newly proposed six-point scale can potentially serve as a valuable tool for aquaculture industry professionals and scientist alike to assess the welfare of teleost fish and evaluate relevant side effects of oil-adjuvanted vaccines in aquaculture. This advancement holds particular significance for marine finfish and European aquaculture, where species like the European sea bass (*Dicentrarchus labrax*) and the gilthead seabream (*Sparus aurata*), two of the most economically important species within the Mediterranean region, are routinely vaccinated with commercial vaccines. Simultaneously, this work also lays the groundwork for further research to be conducted into the implications of contemporary vaccines for farmed fish, expanding the scope to include various factors such as teleost fish species, type of vaccine and adjuvant used, growth rates, final body weight, and the quality of the final products. In conclusion, this showcases the necessity for similar studies to be carried out in order to fully understand the impact of current aquaculture vaccines on aquatic welfare and health. As such, the development of species-specific and tailored scales is essential for enhancing sustainability and promoting humane practices in the sector.

Today, it is widely acknowledged that the mitigation of the side effects of adjuvanted injectable vaccines hinges on optimizing vaccination regimens, refining formulations, and reducing dosage volumes. Strategies like lowering vaccine volume, thereby increasing the antigen density, have shown promise [116]. However, the quest for innovative adjuvants to address the enduring challenge of side effects continues, with nanoparticle-based formulations emerging as a promising breakthrough in aquaculture vaccinology.

## 5. The Promise of Nanoparticle-Based Formulations for the Future

The association between traditional vaccine adjuvants and the adverse post-vaccination side effects on the welfare and health of teleost fish has prompted researchers to explore novel and alternative options, with nanoparticle-based formulations gaining significant traction as the future of this field. As technological expertise progresses, these innovative formulations pave the way for increased vaccination efficacy as novel adjuvants, with the capacity to minimize relevant side effects and adverse reactions. In this section, recent advances in the field of nanoparticle-based adjuvants for aquaculture vaccines will be reviewed, highlighting their position as a potential avenue for ongoing research, where the crossroads of aquatic immunology, aquaculture health management, and technological innovation are met.

### 5.1. Polymeric Nanoparticle Formulations

As already mentioned, polymers represent a significant portion of current nanoparticle-based research attempts focused on vaccine delivery in aquaculture. The shift in scale from microparticles to nanoparticles can offer advantages in immunization enhancement, as smaller-sized particles can activate the immune system more efficiently, triggering both cellular and humoral immune responses in teleost fish. In addition, nanoformulations seem to improve crucial vaccine factors, such as solubility, stability, targeting, permeability, and biocompatibility [127].

PLGA-based nanoparticle applications in aquaculture vaccinology have maintained a prominent presence in the international literature over the past decade, as seen in some of earlier publications [128,129,130,131]. Focusing on more recent studies, PLGA nanoparticles have been utilized for immunization against a diverse range of aquatic pathogens, employing various vaccination technologies and administration methods, while targeting numerous teleost fish species. Dubey et al. have demonstrated that the oral administration of recombinant outer-membrane protein W (OmpW) of *Aeromonas hydrophila* encapsulated in PLGA nanoparticles shows a dose-dependent protective effect in rohu fish (*Labeo rohita*) [132]. Since then, Harshita et al. have also recently shown that the recombinant OmpA can similarly be encapsulated in PLGA nanoparticles and be orally delivered to confer protection in *Aeromonas hydrophila*-challenged zebrafish (*Danio rerio*) [133]. The compatibility of PLGA nanoparticle-based vaccine formulations has also been tested with DNA vaccines against *Aeromonas hydrophila*, both in oral and injectable applications, as seen in the recent works by Alishahi et al. [134,135]. In the case of viral pathogens, a very early first attempt of implementing a PLGA nanoparticle-based oral DNA vaccine against infectious hematopoietic necrosis was made in Adomako’s study [130]; however, recent endeavors have expanded towards more viruses, such as the spring viremia of carp virus (SVCV) and the hemorrhagic septicemia virus (HSV). In a study published in 2019, the efficacy of an inactivated viral vaccine against HSV encapsulated in PLGA nanoparticles was demonstrated by Kole et al. [76], whereas in 2022, Zhang et al. successfully developed a dual-targeting polymer containing PLGA and chitosan nanoparticles to encapsulate a DNA vaccine that was able to confer robust protective immunity in common carp (*Cyprinus carpio*) [136].

The combination of polymers with chitosan nanoparticles as vaccine adjuvants is not coincidental, as chitosan is non-toxic, biodegradable, and has potent mucoadhesive properties that can aid in eliciting strong protective effects upon mucosal immunization in fish [104,137,138]. Several studies combining chitosan nanoparticles with other polymers like alginate and PLGA for various vaccination applications in teleost fish have been published in recent years [139,140,141,142,143,144]. Apart from those, chitosan-complexed nanovaccines have been implemented to counter both bacterial aquatic diseases [145,146,147,148,149,150,151,152,153,154,155,156,157] and viral ones [158,159,160,161,162] in a plethora of new studies. Additionally, novel technologies like chitosan nanoparticles complexed with oxygen nanobubbles have emerged, facilitating the enhanced uptake of vaccines and resulting in superior mucosal immune responses [163]. The synergy of chitosan nanoparticles with traditional adjuvants has also been studied, highlighting that the combination of aluminum adjuvants with chitosan-based nanovaccines can trigger both local and systemic immune responses [164], while on the contrary, Poly I:C’s adjuvanticity appears to deteriorate, presumably due to the delayed, gradual, and stable antigen release that the chitosan particles provide on the microscale [109].

Nevertheless, Collado-González and Esteban highlighted that the inconsistencies in the characterization of the physicochemical properties of chitosan particles, like the degree of acetylation (DA) and the molecular weight (MW), and the different preparation methods used have made it difficult to determine which specific characteristics are most effective in stimulating the teleost fish immune system [106]. This underscores the need for standardizing chitosan nanoparticle-based treatments and improving reporting practices to enhance data comparability and reproducibility. Overall, studies that properly characterized chitosan suggest that low DA (10–15%) and a MW ranging from 50 to 800 kDa may enhance immune gene expression in teleost fish, though the differing amounts of chitosan used and the different immunization regimens followed in different species make direct comparisons difficult at this stage. Collectively, the implementation of polymeric nanoparticle-based vaccine formulations in teleost fish appears promising so far, and there is a pressing need for more studies to explore and correctly assess the advantages of the shift from micro- to nanoscale polymeric materials in aquaculture vaccinology.

### 5.2. Lipid-Based Nanoparticle Formulations

Another type of nanoparticle-based formulation that has gained traction for vaccine delivery and the enhancement of immune responses encompasses lipid nanoparticles, either through nanoliposomes or by using cationic lipid surfactants. These substances typically aid in the stabilization of antigens and can serve as interesting tools for the development of nanoformulations in a wide array of vaccine technologies. Liposomes consist of a hydrophilic core surrounded by hydrophobic bilayers and can be used to encapsulate both hydrophilic and lipophilic compounds [165]. This offers advantages in enhanced solubility, bioavailability, and antigen stability, offering a solid delivery platform that could potentially reinforce immune responses. Alternatively, surfactants like cetrimonium bromide, known as CTAB, are currently being explored for the construction of cationic lipid-based vaccines due to their ability to modify nanoparticle size and surfaces. Additionally, they can alter vaccine charges, therefore opening the gateways for improved delivery, especially through mucosal routes [166,167].

The use of such technologies at the nanoscale are not extensively studied in the field of aquaculture vaccinology; however, there are a few studies that have delved into the use of lipid-based nanoparticle vaccine formulations. In 2019, Ji et al. published a study where nanoliposomes loaded with bacterial LPS and Poly I:C were successfully used to enhance innate immune responses in zebrafish larvae, leading to the upregulation of several immune-related genes and an increase in survival post *Aeromonas hydrophila* challenge following bath immersion vaccination [165]. These results have been succeeded by similar studies, where immunostimulant-loaded nanoliposomes were tested on adult zebrafish, resulting in protection against *Pseudomonas aeruginosa* and SVCV infection both in injection and in immersion applications [168,169]. These results also highlight the recent shift in size from liposome to nanoliposome formulations in teleost fish and pave the way for fish fry vaccination applications by lifting size limitations and improving antigen delivery [165]. A recent development was also made in 2024, where an mRNA-lipid nanoparticle vaccine formulation was successfully applied in the Atlantic salmon (*Salmo salar*), demonstrating that the relatively novel mRNA vaccine technology encapsulated in lipid nanoparticles can indeed be used to express antigens in teleost fish, both in vitro and in vivo [170].

Cationic lipid-based nanovaccines represent the other major part of this category as far as nanoparticle-based formulations in aquaculture vaccinology are concerned. Despite the lack of reports on teleost fish applications, recent years have witnessed the emergence of a few studies implementing this technology to develop mucoadhesive immersion nanovaccines against bacterial pathogens. In Thangsunnan et al.’s published study, a mucoadhesive nanovaccine against *Francisella noatunensis* subsp. *orientalis* was successfully constructed using CTAB as a surfactant [167]. This formulation conferred protection comparable to that of a whole-cell inactivated vaccine, inducing strong immune responses after boost immunization. Gene expression analysis revealed a significant upregulation of both IgM and IgT in vaccinated fish, confirming enhanced mucosal and systemic responses in red tilapia (*Oreochromis* sp.). Similarly, Bunnoy et al. developed a vaccine of similar technology to combat columnaris disease [168]. The nanovaccine effectively targeted the mucosal-associated lymphoid tissues (MALT) in the skin and gills of the Asian seabass (*Lates calcarifer*), resulting in a strong immune response, where vaccinated fish showed higher antibody levels and survival rates (65.83–72.50%) compared to the controls. This formulation stimulated both innate and adaptive immunity, resulting in an increased expression of immune-related genes like IgM and MHC-IIα. However, while effective, the vaccine was linked with early mortality, indicating that further optimization is needed in vaccination protocols.

Below, we present a table (Table 4) that collectively summarizes the efficacy of lipid-based nanoparticle vaccine formulations discussed above. Special reference is given to the teleost fish species studied, the type of lipid-based nanoparticle technology implemented, the pathogen species targeted, the vaccine technology used, the method of administration, and the immunological efficacy reached in these studies following vaccination. This summary provides a unified approach, showcasing the potential applicability of different approaches across a diverse range of aquaculture contexts, despite lipid-based nanoparticle technologies being in their early stages as far as fish vaccinology is concerned.

### 5.3. Carbon Nanotubes and Inorganic Nanoparticle Formulations

Lastly, carbon nanotubes (CNTs) represent a cutting-edge paradigm with regard to nanoparticle-based vaccine formulations for teleost fish, offering an alternative tool to enhance the delivery and immunogenicity of aquaculture vaccines, particularly through mucosal routes. Carbon nanotubes comprise hollow, cylindrical structures made from rolled up graphene sheets. These nanotubes can either include a single, or many graphene layers, making the distinction between single-walled carbon nanotubes (SWCNTs) and multi-walled carbon nanotubes (MWCNTs) [171].

Carbon nanotubes are considered inert and not immunogenic by themselves, having relatively low toxicity and high biocompatibility [172]. A recent study conducted by Cimbaluk et al. demonstrated that this nanomaterial does not induce any genotoxic effects like single or double DNA strand breaks in teleost fish erythrocytes. However, CNT-DNA crosslinks were observed, alluding to CNTs preventing the separation of DNA strands and potentially compromising the process of replication [173]. Chronic exposure to this material has indeed been linked to the induction of oxidative stress, pollutant accumulation, neurotoxicity, inflammation, apoptotic signaling, and histological pathologies in fish [173,174,175,176,177,178,179,180,181]. Nonetheless, the relevance of these studies to vaccination regimens remains to be elucidated and warrants further research to be conducted, especially since the physicochemical properties of the CNTs appear to make a difference in their toxicity according to Jiang et al. [182]. The modification of CNTs with other polymers also appears to lower toxicity claims, as is the case with chitosan grafting in Wisdom et al.’s study [183], which underpins the possibility of an ideal formulation to be discovered soon through the combination of nanopolymeric materials.

The functionalization of CNTs for immunization applications in teleost fish typically shows reduced toxicity and can have profound effects on the stimulation of immune cells [184]. During the past decade, several studies have accumulated in the international literature dealing with CNT-based vaccination in a wide range of species, yet the focus seems to be particularly set in immunization applications against viral aquatic diseases. Nevertheless, there have been a few studies showcasing that CNTs can be used to combat bacterial diseases as well, with *Aeromonas hydrophila* centered as the main pathogen of interest. In 2015, Gong et al. developed a novel formulation using SWCNTs to deliver a recombinant vaccine against the pathogen in juvenile grass carp (*Ctenopharyngodon idellus*) [185]. The vaccine, administered via both injection and immersion, led to a higher antibody production and a significant upregulation of immune markers, achieving similar survival rates to traditional injection methods. A year later, the same group published another study, highlighting the upregulation of immune-related genes, which was induced by their SWCNT-based vaccine compared to a SWCNT-free control [186]. More recent efforts were reported in 2020, with Zhang et al. successfully demonstrating the immunoprotective effect of a whole-cell lysed inactivated vaccine, utilizing SWCNTs as a carrier [187]. In 2024, Cao et al. shifted the focus by publishing an interesting study in which they constructed a CNT nanocarrier immersion vaccine encoding the immunogenic recombinant protein Sip [188]. This vaccine formulation promoted the expression of immune genes, induced relatively high serum antibody production, and enhanced related enzyme activities, ultimately conferring significant cross-protection against *Streptococcus agalactiae* and *Streptococcus iniae* in red tilapia.

As previously mentioned, however, the main body of work revolving around CNT-based vaccination in teleost fish stems primarily from applications dealing with viral diseases. The grass carp reovirus (GCRV) constitutes a prime example, since there are several publications documenting the efficacy of CNT-based nanovaccines in a plethora of different vaccine technologies, such as recombinant subunit vaccines [189,190,191] and DNA vaccines [192,193,194]. Another grass carp virus, the spring viraemia carp virus (SVCV), has similarly garnered the attention of CNT-based vaccination applications, as evidenced by recent studies [195,196,197,198,199]. Zhao et al. have successfully constructed and demonstrated the effect of a SWCNT-based subunit vaccine against the infectious spleen and kidney necrosis virus (ISKNV) in mandarin fish (*Siniperca chuatsi*) [200,201], and the same strategy was implemented for a DNA vaccine [202]. The most recent publications from this group are currently showcasing the efficacy of a mannose-modified CNT subunit vaccine designed to target antigen-presenting cells both in injection and in immersion formats [203,204]. Apart from ISKNV, CNT-based vaccination has been reported to be efficient against another iridovirus, named the grouper iridovirus of Taiwan (TGIV) [205,206], as well as other viral families, such as herpesviruses [207,208], rhabdoviruses [209], betanodaviruses [210], and the recently classified largemouth bass ulcerative syndrome ranavirus [211]. An indicative table (Table 5) summarizing the recent studies of CNT-based vaccination studies in teleost fish mentioned can be read below:

Staying within the context of nanotube formulations, it is worth noting that an inorganic alternative of CNTs has recently emerged in the realm of aquaculture vaccines, offering an alternative to its organic relative and addressing the gap in the literature regarding the use of inorganic nanoparticle-based formulations in teleost fish [212]. Halloysite nanotubes (HNTs) are naturally occurring tubular clay structures similar to CNTs, featuring a unique disparity in charge between their inner and outer surfaces [213]. They provide an environmentally safe and cost-efficient platform for vaccine delivery throughout the gastrointestinal tract, allowing for the encapsulation of susceptible antigens and their subsequent release in a pH-dependent manner, as well as for their modification with other materials to enhance their properties [213,214].

Pumchan et al. have recently harnessed the power of HNTs to test out and develop a novel oral nanovaccine delivery system to combat streptococcosis in tilapia (*Oreochromis* sp.) [214]; remarkably, they found that chitosan-modified HNTs demonstrated strong resilience to stomach acidity, bypassing gastric digestion and delivering a whole-cell inactivated vaccine to the intestines that promoted an immune response via the gut-associated lymphoid tissues (GALTs). This formulation effectively induced immunity against *Streptococcus agalactiae* serotype Ia, exhibiting similar disease protection and survival rates when compared to traditional injectable vaccines. The chitosan coating of HNTs enhanced the immune response and adherence to intestinal cells, positioning this nanoparticle-based formulation as a promising candidate for efficacious oral vaccination. Taken together with Zhang et al.’s recent publication on the use of a mesoporous silica nanoparticle-based delivery system for oral vaccination against *Vibrio alginolyticus* [215], these few studies highlight the use of inorganic nanoparticles in teleost fish immunization, ultimately completing the puzzle of a much promising arsenal for the future of adjuvant research in aquaculture vaccinology.

## 6. Conclusions

Adjuvants are quintessential elements of modern aquaculture vaccines designed for teleost fish, as they aid in inducing potent and long-lasting immune responses that can protect reared species against aquatic diseases. Progress made in understanding the exact mechanisms of action behind various adjuvants has facilitated the development of many innovative and novel vaccine formulations, designed specifically to induce desired immunization outcomes. Combined with recent insights into aquatic and teleost fish immunology, these advancements have enabled the introduction of progressively more sophisticated vaccine and adjuvant technologies to be implemented in aquaculture settings, indicating that the future of vaccinology research and vaccine development in one of the fastest growing sectors is an excellent field for investment and exploration.

Despite vaccine adjuvants forming an integral part of aquaculture vaccines, there have been growing concerns about their potential adverse effects in teleost fish health, raising questions regarding product quality assurance and associated welfare issues in modern aquaculture. The use of contemporary vaccine adjuvants, such as oil-based emulsions, has been linked with side effects like tissue and organ adhesions, inflammatory reactions, granulomas, and spatial discolorations at the sites of injections, since intraperitoneal injection is currently the most widely used method of vaccine administration in the field. These findings have led researchers and aquaculture professionals alike to explore alternative ways of teleost fish immunization, in an effort to minimize cost, labor, and potential side effects, while maximizing the immunization outcomes. Apart from the shift in the administration route, as current trends dictate, the exploration of novel adjuvants offers another backroad for circumnavigating the limitations of current aquaculture vaccines, providing cutting-edge solutions to persisting issues, such as impotent immunization and compromised fish welfare. Taken together, these two elements can significantly improve the sustainability of the aquaculture industry and facilitate the establishment of more welfare-friendly practices that will ensure the quality of aquatic food products in accordance with the consumer demands.

This is particularly true for mucosal routes of vaccination, such as oral or immersion vaccination, where the “depot” effect observed in injectable vaccines is typically absent or significantly reduced. Unlike traditionally adjuvanted injectable vaccines, which provide a localized, prolonged release of antigens, vaccine administration through mucosal routes often lacks the advantage of concentrated antigen delivery. This can lead to shorter durations of immune responses and reduced vaccination efficacy in teleost fish. Consequently, these methods are presently better suited for booster vaccination applications in today’s aquaculture practice. Addressing this issue requires innovative delivery technologies, like encapsulation or slow-releasing technologies, as well as novel adjuvants with potent mucoadhesive or mucosal-homing properties that can simulate depot-like behavior by prolonging antigen exposure and enhancing immune stimulation.

Currently, the spearhead of such attempts, as far as modern innovation is concerned, lies in the utilization of nanoparticle-based formulations as a means of replacing or improving the traditional vaccine adjuvants in aquaculture vaccinology. By harnessing the power of technological advances and biological knowledge, the utilization of nanoparticles can enhance vaccine delivery, potentiate immune responses, and ultimately confer superior protective effects in reared species, improving vaccine formulations and mitigating relative side effects linked to conventional vaccine adjuvants. Polymeric nanoparticles, lipid-based nanoformulations, and nanomaterials of organic or inorganic origin are at the forefront of current research endeavors employing nanoparticle-based formulations for immunization purposes in teleost fish. However, even though they appear promising, these applications are still considered to be in their relative infancy, as most of these technologies have only been tested in fish for no more than a decade. Ultimately, this warrants further research efforts to be put into assessing the application of these materials, so that concrete and relative evidence will be accumulated on their efficacy, optimization, and safety in aquatic species.

Overall, the assessment of animal safety and welfare should be placed as a top priority in trials exploring novel formulations, as this can potentially ensure faster application approval and the establishment of welfare-friendly vaccination practices in the sector. The need for tools and attention to effectively monitor and evaluate teleost fish welfare is pressing, especially in the realm of aquaculture immunization, as many different technologies, employing various administration routes in several species, are currently being utilized globally, in a plethora of aquaculture sites. A series of properly standardized technology- and species-specific guidelines can potentially help in establishing a framework where vaccine adjuvants and their effects can be tracked, compared, and evaluated on an evidential basis, in an effort to accelerate vaccine development and sort out potential candidates for novel vaccine formulations. By striking a balance between vaccination efficacy and teleost fish welfare, the next generation of aquaculture vaccines will probably be soon available in the mass market, ushering in a new era for the future of the aquaculture industry.

## Figures and Tables

**Table 1:** A cumulative summary of the three major pattern-recognition receptor (PRR) types identified in teleost fish according to the current and up-to-date literature. It is evident that the teleost fish species is an important parameter in the presence or absence of certain PRRs, as well as in their associated ligands. Homologues of mammalian PRRs have been characterized in a plethora of teleost fish species. This table includes some indicative ones for reference [26,27,28,29,30,31,32,33,34,35,36,37,38,39,40,41].

Toll-like Receptors (TLRs)		
Receptor	Associated Ligands	Indicative Teleost Fish Species
TLR1	Lipopeptides	Rainbow Trout, Large Yellow Croaker, Carp, Pufferfish, Orange-Spotted Grouper, European Sea Bass, Turbot
TLR2	Lipopeptides, PGN, LTA, Pam_3_CSK_4_	Carp, Catfish, Orange-Spotted Grouper, European Sea Bass, Turbot, Gilthead Seabream
TLR3	dsRNA, poly(I:C)	Carp, Pufferfish, Zebrafish
TLR4	Unknown	Carp, Catfish, Rare Minnow, Zebrafish
TLR5	Flagellin	Atlantic Salmon, Japanese Flounder, Channel Catfish, Gilthead Seabream, Rainbow Trout, Pufferfish, Zebrafish, Turbot
TLR7	ssRNA	Channel Catfish, Grass Carp, Pufferfish, Rainbow Trout, Zebrafish, Turbot
TLR8	ssRNA	Atlantic Salmon, Channel Catfish, Pufferfish, Rainbow Trout, Turbot
TLR9	CpG motifs	Atlantic Salmon, Cobia, Japanese Flounder, Rainbow Trout, Zebrafish, European Seabass, Gilthead Seabream, Turbot
TLR13	rRNA	Atlantic Salmon, Channel Catfish, Orange-Spotted Grouper
TLR14	Unknown	Japanese Flounder, Orange-Spotted Grouper, Pufferfish
TLR18	Unknown	Channel Catfish, Grass Carp, Zebrafish
TLR19	dsRNA	Channel Catfish, Grass Carp, Zebrafish
TLR20	Unknown	Carp, Channel Catfish, Zebrafish
TLR21	CpG motifs	Channel Catfish, Grass Carp, Orange-Spotted Grouper, Zebrafish, Turbot
TLR22	dsRNA, poly(I:C)	Atlantic Cod, Channel Catfish, Grass Carp, Pufferfish, Zebrafish, European Seabass, Turbot, Gilthead Seabream
TLR23	Unknown	Atlantic Cod, Pufferfish
TLR25	Unknown	Channel Catfish, Fathead Minnow, Nile Tilapia
TLR26	LPS, poly(I:C)	Channel Catfish, Yellow Catfish
TLR28	LPS, poly(I:C)	Brown Croaker
**NOD-like Receptor (NLR) Superfamilies**		
NLR-A	LPS, PGN (iE-DAP, MDP), Poly (I:C)	Grass Carp, Nile Tilapia, Rainbow Trout, Channel Catfish, Zebrafish
NLR-B	Unknown	Grass Carp
NLR-C	Unknown	Grass Carp, Brown Croaker
**RIG-1-like Receptors (RLRs)**		
RIG-1	dsRNA	Zebrafish
MDA5	dsRNA	Grass Carp
LGP2	dsRNA	Grass Carp

**Table 2:** Commercially available Montanide^TM^ adjuvants intended for use in teleost fish according to the manufacturer, at the time of writing. The vast majority are based on mineral oils and are intended for use via injection vaccination.

Currently Available Montanide^TM^ Adjuvants for Use in Aquaculture Vaccinology		
Series Name	Technology	Route
Montanide^TM^ ISA 61 VG	Mineral Oil base for W/O Emulsions	Injection
Montanide^TM^ ISA 50 V2	Mineral Oil base for W/O Emulsions	Injection
Montanide^TM^ ISA 70 VG	Mineral Oil base for W/O Emulsions	Injection
Montanide^TM^ ISA 71 VG	Mineral Oil base for W/O Emulsions	Injection
Montanide^TM^ ISA 71 R VG	Mineral Oil base for W/O Emulsions	Injection
Montanide^TM^ ISA 761 VG	Mineral Oil base for W/O Emulsions	Injection
Montanide^TM^ ISA 78 VG	Mineral Oil base for W/O Emulsions	Injection
Montanide^TM^ ISA 763B VG	Non-Mineral Oil base for W/O Emulsions	Injection
Montanide^TM^ ISA 660 VG	Non-Mineral Oil base for W/O Emulsions	Injection
Montanide^TM^ GR	Oil base for W/O Emulsions containing a Protective Matrix	Oral
Montanide^TM^ IMS 1312 VG	Combination of Micro-Emulsions with an Immunostimulating Compound	Immersion

**Table 3:** A side-by-side adaptation comparison between Spielberg’s original scale for assessing the Atlantic salmon post-furunculosis vaccination and the newly proposed scale for assessing vaccination side effects in the European seabass by Tziouvas and Varvarigos.

Score (0–6)	Spielberg Scale [126] (Atlantic Salmon)	Tziouvas and Varvarigos Scale [125] (European Sea Bass)
**0**	No visible lesions.	No adhesions or peritoneal lesions. Fish with this score may be unvaccinated by mistake.
**1**	Very slight adhesions, usually localized near the injection site, with minimal peritoneal opacity post evisceration. Unlikely to be noticeable by laypeople.	Soft, localized adhesions around organs, mainly the intestine; the swim bladder and the abdominal wall are unaffected. Organs separate easily, and nodules or lesions are absent. Typically seen soon post vaccination.
**2**	Same as the score above, but with minor adhesions connecting the colon, spleen, or caudal pyloric caeca to the abdominal wall. Peritoneum remains slightly opaque after adhesion removal.	Soft, localized adhesions affecting some organs, but sparing the swim bladder and the abdominal wall. Few small, non-pigmented granulomatous nodules are present.
**3**	Moderate adhesions extending to more cranial organs, like the pyloric caeca, liver, or ventricle, attaching them to the abdominal wall. Minor lesions visible post evisceration, removable manually.	Widespread and soft adhesions involving multiple organs, but not the swim bladder or the abdominal wall. Small, mostly non-pigmented nodules are frequent and under 1 mm in diameter.
**4**	Major adhesions with granulomas and extensively connected internal organs appearing as a single unit. Moderate lesions may be difficult to remove manually. Likely noticeable by laypeople during evisceration.	Soft to moderately hard widespread adhesions, affecting the swim bladder and the abdominal wall. Separation requires moderate force, with small and some larger nodules (up to 2 mm), mostly non-pigmented, but some pigmented.
**5**	Extensive lesions affecting nearly all internal organs. Peritoneum is thickened and opaque, with prominent, pigmented lesions on the fillet. Visible damage remains after lesion removal. It is likely to be noticed by laypeople during evisceration.	Moderately hard, widespread adhesions involving the swim bladder and the abdominal wall. Moderate force is needed to separate organs, risking rupture. Numerous nodules from pin-point to >4 mm, non-pigmented and pigmented.
**6**	Very pronounced lesions, often with significant melanization. Viscera cannot be removed without damaging fillet integrity, leaving substantial carcass damage.	Strong adhesions connecting visceral and parietal peritoneum, with numerous nodules exceeding 8 mm. Organ separation risks rupture; no fillet lesions, but potential growth impacts are possible.

**Table 4:** Recent studies on teleost fish vaccinations utilizing lipid nanoparticle-based formulations. So far, the two approaches implemented in in vivo vaccination trials mainly include encapsulating nanoliposomes and cationic lipid-based nanoemulsions.

Vaccine Technology	Delivery Route	Pathogen (Antigen)	Teleost Species	Efficacy	Source
Encapsulating Nanoliposomes	Immersion	*Aeromonas hydrophila* (*E. coli* LPS + Poly I:C)	Zebrafish (*Danio rerio*)	Increased survival rate post challenge and upregulation of immune-related genes	[165]
CTAB-Cationic Lipid Nanoemulsion	Immersion	*Flavobacterium oreochromis* (Formalin-Inactivated)	Asian Seabass (*Lates calcalifer*)	Increased survival rate post challenge and upregulation of immune-related genes	[166]
CTAB-Cationic Lipid Nanoemulsion	Immersion	*Francisella noatunensis* subsp. *Orientalis* (Formalin Inactivated)	Red Tilapia (*Oreochromis* sp.)	Reduced bacterial load in tissues, enhanced antibody titers, and upregulation of immune-related genes	[167]
Encapsulating Nanoliposomes	Injection and Immersion	*Pseudomonas aeruginosa* and SVCV (*E. coli* LPS + Poly I:C)	Zebrafish (*Danio rerio*)	Increased survival rates against both bacterial and viral challenges	[169]

**Table 5:** A summary of recent carbon nanotube-based applications in teleost fish vaccinology, based on the recent literature. Findings suggest that CNT-based vaccination aligns predominantly with two underutilized vaccine technologies in commercial applications, recombinant subunit, and DNA vaccine platforms, indicating a potential shift in the future of aquaculture vaccines thanks to nanoparticle formulations.

CNT Type	Vaccine Technology	Delivery Route	Pathogen (Antigen)	Teleost Species	Source
Single-Walled	Recombinant Subunit	Intramuscular Injection and Bath Immersion	*Aeromonas hydrophila* (aerA)	Grass Carp	[185]
Single-Walled	DNA Vaccine	Intramuscular Injection	*Aeromonas hydrophila* (aerA)	Grass Carp	[186]
Single-Walled	Whole-cell Inactivated Vaccine	Intraperitoneal Injection and Bath Immersion	*Aeromonas hydrophila* (Bacterial Lysate)	Grass Carp	[187]
Single-Walled	Recombinant Subunit	Bath Immersion	*Streptococcus* sp. (rSip)	Tilapia	[188]
Single-Walled (Mannose Modified)	Recombinant Subunit	Bath Immersion	GCRV (VP7)	Grass Carp	[189]
Single-Walled	Recombinant Subunit	Bath Immersion	GCRV (VP7)	Grass Carp	[190]
Single-Walled	Recombinant Subunit	Bath Immersion	GCRV (VP4-3)	Grass Carp	[191]
Single-Walled	DNA Vaccine	Intramuscular Injection and Bath Immersion	GCRV (VP4-3, VP5, VP7)	Grass Carp	[192,193,194]
Single-Walled	DNA Vaccine	Intramuscular Injection	SVCV (M)	Common Carp	[195]
Single-Walled	Recombinant Subunit	Intramuscular Injection and Bath Immersion	SVCV (G)	Common Carp	[196]
Single-Walled	DNA Vaccine	Intramuscular Injection and Bath Immersion	SVCV (G)	Common Carp	[197]
Single-Walled	DNA Vaccine	Bath Immersion	SVCV (M)	Common Carp	[198]
Single-Walled (Mannose Modified)	Recombinant Subunit	Bath Immersion	SVCV (G)	Common Carp	[199]
Single-Walled	Recombinant Subunit	Bath Immersion	ISKNV (MCP)	Mandarin Fish	[200,201]
Single-Walled	DNA Vaccine	Bath Immersion	ISKNV (MCP)	Mandarin Fish	[202]
Single-Walled (Mannose Modified)	Recombinant Subunit	Intramuscular Injection and Bath Immersion	ISKNV (MCP)	Mandarin Fish	[203,204]
Single-Walled	Recombinant Subunit	Bath Immersion	TGIV (MCP, P2)	Pearl Gentian Grouper	[205,206]
Single-Walled	DNA Vaccine	Intramuscular Injection and Bath Immersion	KHV (ORF 149)	Koi Fish	[207,208]
Single-Walled	Recombinant Subunit	Bath Immersion	MSRV (G)	Largemouth Bass	[209]
Single-Walled	Recombinant Subunit	Bath Immersion	NNV (MCP)	Pearl Gentian Grouper	[210]
Single-Walled	Recombinant Subunit	Bath Immersion	LBUSV (MCP)	Largemouth Bass	[211]
